# Serious illness communication skills training for emergency physicians and advanced practice providers: a multi-method assessment of the reach and effectiveness of the intervention

**DOI:** 10.1186/s12904-024-01349-y

**Published:** 2024-02-21

**Authors:** Oluwaseun Adeyemi, Alexander D. Ginsburg, Regina Kaur, Allison M. Cuthel, Nicole Zhao, Nina Siman, Keith S Goldfeld, Lillian Liang Emlet, Charles DiMaggio, Rebecca Liddicoat Yamarik, Jean-Baptiste Bouillon-Minois, Joshua Chodosh, Corita R. Grudzen, The PRIM-E. R. Investigators, Lauren T. Southerland, Peg Gulker, Andrew Johnston, Arvind Venkat, David Chuirazzi, John O’Neill, Kelly Szabo, Rachel Urosek, Ashley Deutsch, Elizabeth Schoenfeld, Melissa Shaw, Tricia Guerino, Alayna Perko, Lauren Cameron- Comasco, Michael Banish, Pamela Sloan, Robert Swor, Ronny Otero, Aaron Elliot, Kim Reiner, Nicole Hurd, Brittany Ballaron, Kei Ouchi, Natasha Egorova, Andrew Dundin, Niza Troncoso, Robin Powell, Barbara J. Debbage, Deborah Johnson, John Powell, Julie Cooper, Doretha Graham-Brekke, Erin Zimny, Glenn Tokarski, Joseph Miller, Olive Sadia, Christopher Richardson, Jennifer Kroll, Jennifer Siller, Jessica Fleischer-Black, Karen Evelyn, Laura Stark, Lauren Gordon, Lynne Richardson, Michelle Lin, Audrey Tan, Alicia Sommer, Caitlin Loprinzi-Brauer, Heather Heaton, Laura Walker, MFernanda Bellolio, Molly Christenson, Donna Shelley, Audie Liametz, Barry Rosenthal, Ian Wittman, Kathy Peterson, Lila Hageman-Sheehan, Rajneesh Gulati, Robert Smeltz, Staci Mandola, Stephen Stark, Suchismita Datta, Susan Cohen, Tisha Thompson, Katharine Lawrence, Abraham A. Brody, Leora Horwitz, Nicholas Genes, Ashley Shreves, Deidre Bolden, Kelly Hutchinson, Maureen Gang, Rebecca Goett, Sangeeta Lamba, Eric Isaacs, Jennifer Harris, Karen Martinez, Matthew Shaw, Rebecca Murray, Rosemarie Fernandez, Shannon Bledsoe, Travis Wood, Matthew Ryan, Benjamin S. Abella, Elizabeth Long, Gabriela De Hoyos, Julie Uspal, M. Bradley Falk, Phillip Landis, Ahmed Elsayem, Cecilia Yniguez, Danielle Milling, Denise Langabeer, Sorayah Bourenane, Terri Cridge, Troy Madsen, Emilia Boutsioulis, Hannah Nofsinger, Karen Jubanyik, Theresa Cohen, Marie-Carmelle Elie

**Affiliations:** 1 Ronald O. Perelman Department of Emergency Medicine, NYU Grossman School of Medicine, New York, 227 East 30thStreet, New York, NY 10016 USA; 2 Department of Emergency Medicine, Mayo Clinic, Rochester, MN USA; 3 CHI Saint Joseph Health, London, KY USA; 4 Renaissance School of Medicine, Stony Brook University, Stony Brook, NY USA; 5 Department of Population Health, NYU Grossman School of Medicine, New York, NY USA; 6 Department of Critical Care Medicine, University of Pittsburgh, Pittsburgh, PA USA; 7 Department of Surgery, NYU Grossman School of Medicine, New York, NY USA; 8 Veteran Affairs Long Beach Healthcare System, Long Beach, CA USA; 9 Emergency Department, CHU Clermont-Ferrand, Clermont-Ferrand, France; 10 Department of Medicine, NYU Grossman School of Medicine, New York, NY USA; 11 Veteran’s Affair, New York Harbor Healthcare System, New York, NY USA; 12 Department of Medicine, Memorial Sloan Kettering Cancer Center, New York, NY USA

**Keywords:** Palliative care, Emergency medicine, Serious illness conversation, VitalTalk, Education and training

## Abstract

**Background:**

EM Talk is a communication skills training program designed to improve emergency providers’ serious illness conversational skills. Using the Reach, Effectiveness, Adoption, Implementation, and Maintenance (RE-AIM) framework, this study aims to assess the reach of EM Talk and its effectiveness.

**Methods:**

EM Talk consisted of one 4-h training session during which professional actors used role-plays and active learning to train providers to deliver serious/bad news, express empathy, explore patients’ goals, and formulate care plans. After the training, emergency providers filled out an optional post-intervention survey, which included course reflections. Using a multi-method analytical approach, we analyzed the reach of the intervention quantitatively and the effectiveness of the intervention qualitatively using conceptual content analysis of open-ended responses.

**Results:**

A total of 879 out of 1,029 (85%) EM providers across 33 emergency departments completed the EM Talk training, with the training rate ranging from 63 to 100%. From the 326 reflections, we identified meaning units across the thematic domains of improved knowledge, attitude, and practice. The main subthemes across the three domains were the acquisition of Serious Illness (SI) communication skills, improved attitude toward engaging qualifying patients in SI conversations, and commitment to using these learned skills in clinical practice.

**Conclusion:**

Our study showed the extensive reach and the effectiveness of the EM Talk training in improving SI conversation. EM Talk, therefore, can potentially improve emergency providers’ knowledge, attitude, and practice of SI communication skills.

**Trial registration:**

Clinicaltrials.gov: NCT03424109; Registered on January 30, 2018.

**Supplementary Information:**

The online version contains supplementary material available at 10.1186/s12904-024-01349-y.

## Introduction

More than half of seriously ill older adults visit the Emergency Department (ED) in the last six months of life [1, 2]. It is estimated that between 50 and 60 percent of seriously ill older adults do not have advanced directives [3, 4] and are at risk of receiving care inconsistent with their wishes [5]. The ED presents an opportunity to engage these patients in discussions focused on goals of care, advanced directives, and willingness to obtain hospice and palliative care. Initiating serious illness (SI) conversations are never easy for providers, irrespective of specialty [6–8]. Emergency Medicine (EM) providers tend to avoid such conversations as they are more likely to assume that they are better suited to provide life-prolonging interventions and providers of other specialties are better equipped to handle such conversations [9].

Unlike medical specialties with a controlled patient-provider environment like primary care and oncology, navigating SI conversations in the ED environment requires additional skills in engaging patients and caregivers in a fast-paced environment while maintaining patient privacy. EM Talk, adapted from VitalTalk, [10, 11] is the only known SI communication skill training model available for EM providers. It is unknown how effective the educational intervention is in improving the knowledge, attitude, and practice of EM providers. However, other specialty-focused adaptations of VitalTalk such as OncoTalk (for oncology providers) [12, 13] had been associated with a substantial increased skill acquisition in delivering bad news and transitioning qualifying patients to palliative care [14, 15]. Also, Geritalk for geriatric providers [16, 17] , has been associated with substantial improvement in self-reported preparedness and practice of engaging in SI conversations [18, 19]. Integral to the VitalTalk training framework are evidence-based pedagogical techniques such as the use of simulated patients and caregivers, role-playing, and small group learning [15, 17, 20]. It is, therefore, plausible that EM Talk may exhibit similar effectiveness as Geritalk and OncoTalk.

Understanding the reach and effectiveness of EM Talk is important as it may provide the necessary Accreditation Council of Graduate Medical Education competency in engaging in SI conversations [21]. Hence, to evaluate the reach and effectiveness of the EM Talk, we adopted the Reach, Effectiveness, Adoption, Implementation, and Maintenance (RE-AIM) framework [22–24]. The two-decade-old RE-AIM framework is a planning and evaluation tool commonly used to assess project implementation across clinical, public health, and health behavior-focused research [22]. For this study, we focused on assessing the intervention’s reach – defined as the absolute number of persons who participated in the intervention, and the intervention’s effectiveness – defined as the impact of the intervention on individual outcome measures [22]. Therefore, the aim of this study, is to assess the reach of EM Talk and its effectiveness in improving knowledge, attitude, and practice among EM providers.

## Methods

### Study design

We employed a multi-method approach to assess the reach and effectiveness of the EM Talk intervention in providing SI communication skills for full-time EM physicians and advanced practice providers (hereafter referred to as EM providers). The advanced practice providers involved in SI communication skills training were those involved in the care of high-acuity patients. Consistent with this multi-method research design, [25] the reach of the intervention was assessed quantitatively using a cross-sectional study design while the effectiveness was assessed qualitatively using a conceptual content analytical design [22]. We defined the reach of the EM Talk intervention as the absolute number and proportion of representative EM providers across each participating ED that obtained the SI communication skill training. Also, we estimated the number of seriously ill patients encountered by the trained EM providers and the estimated yearly number of patients each trained provider will reach across each ED, assuming a 100 percent practice rate. We defined the effectiveness of the EM Talk intervention as the self-reported thematic domains of improved knowledge, attitude, and practice of SI communication skills. The unit of analysis of the quantitative study was at the institutional level while the unit of analysis of the qualitative study was phrases and sentences. This study followed the consolidated criteria for reporting qualitative research (COREQ) guideline [26].

### Study population

The study population was a census of full-time EM providers across 33 EDs enrolled in the Primary Palliative Care for Emergency Medicine (PRIM-ER) study. The PRIM-ER study is a cluster-randomized pragmatic trial that assesses the impact of EM provider interventions on healthcare utilization and outcomes among seriously ill older adults that visit the ED [27]. The PRIM-ER intervention consists of (1) education in palliative and end-of-life care for EM providers and emergency nurses, (2) communication skills training and simulation workshops for EM providers (using EM Talk training) and emergency nurses (using the End-of-Life Nursing Education Consortium (ELNEC) training), and (3) the integration of a clinical decision support tool to identify and engage seriously ill older adults in SI conversations. The PRIM-ER study is still ongoing and this index study explores the implementation of the intervention by reporting the reach and effectiveness of the intervention. This study does not provide reports across the study arms or an assessment of the primary outcomes of the PRIM-ER study. We had reported the reach of the ELNEC intervention and emergency nurses' perceived barriers and solutions to conducting SI conversations in the ED [28]. The current study focuses on the reach and effectiveness of EM Talk across the cross-section of providers who underwent the training.

### EM Talk intervention

EM Talk is a one-day 4-h SI communication skills training session, delivered both in-person and virtually. Consistent with our cluster-randomized stepped wedge design, [27] the EM Talk training occurred sequentially across 33 EDs for three years (2019 to 2021). Before each training session, an EM Champion – an influential EM provider, was selected to encourage and mobilize EM providers for the training and organize the training logistics in their ED. The first half of the session comprised large group lectures and the second half of the session focused on small group practice for delivering bad news, discussing goals of care, and for reflective exercises. Each session was facilitated by two VitalTalk-trained personnel. Details of the EM Talk course description have been published earlier [20]. Within one week after the SI communication skill training, EM providers completed a self-administered post-training survey and received a five-unit continuing medical education (CME) credit and a $67 gift card for their time.

### Quantitative data analysis

We obtained administrative data from each ED of the PRIM-ER study and the Centers for Medicare & Medicaid Services (CMS). Using the administrative data, we computed the counts of the EM providers that completed the EM Talk training and generated the sum of EM providers in each participating ED. Using the CMS data, we generated the yearly number of seriously ill patients that visit the ED by computing the mean of the number of qualifying seriously ill patients that visited the participating sites between 2018 and 2020. A qualifying seriously ill patient is a patient, 66 years or older, that visited the ED within the study period with a life-limiting illness identified using a GAGNE index (a measure of one-year mortality) greater than 6 [27, 29]. We defined the proportion of EM providers trained as the number of EM providers trained divided by the total number of EM providers in the participating EDs (Table 1). We defined the estimated number of seriously ill patients as the yearly average number of qualifying seriously ill patients in each ED multiplied by the proportion of EM Talk-trained providers in the ED. We defined the yearly seriously ill patient and EM provider ratio as the average yearly average of the seriously ill patients reached divided by the number of EM providers trained.

**Table 1:** Statistical definitions of the reach of the primary palliative care for emergency medicine (PRIM-ER) Study

Term	Statistical Definitions
Seriously ill (SI) patient	Meets the following criteria: a. 66 years and older b. Visited one of the 33 EDs at least once c. Has a GAGNE > 6
Proportion of EM providers trained	$\frac{Total\;Number\;of\;EM\;Providers\;that\;Completed\;EM\;Talk\;Training}{Total\;Number\;of\;EM\;Providers\;in\;Participating\;ED}$
Yearly average of SI patient visits	$\frac{Total\;Number\;of\;SI\;patients\;that\;had\;unique\;ED\;visits\;between\;2018\;and\;2020}{3\;(the\;number\;of\;years)}$
Estimated number of SI patients reached	$Yearly\;average\;of\;unique\;SI\;patients\;that\;visited\;the\;33\;EDs\ast Proportion\;of\;EM\;providers\;trained$
SI patient: EM provider ratio	$\frac{Estimated\;number\;of\;SI\;patients\;reached}{Proportion\;of\;EM\;providers\;trained}$

### Qualitative data analysis

Consistent with a conceptual content analytical approach, we identified codes that fell into three a priori-defined thematic domains of improved knowledge, attitude, and practice. The knowledge, attitude, and practice (KAP) theory [30], a commonly used theoretical model to assess behavior change, divides the steps in behavioral change into knowledge acquisition, belief and attitude generation, and practice creation. We selected the KAP theory as the conceptual model to assess the effectiveness of the EM Talk intervention since the intent of the intervention was to equip EM providers with communication skills (knowledge acquisition), create a simulated practice experience (attitude generation) so that they can effectively engage seriously ill patients on discussions around goals of care (practice creation).

Data for the qualitative analysis was from one of the open-ended questions in the EM Talk post-training survey – designed consistent with the requirement of continuing medical education assessment (Appendix 1) [31]. The specific question selected for this qualitative study was: *In the space below, please reflect on your personal experience with this educational intervention.* Responses that were collected before, during, and after the peak period of the COVID-19 pandemic were prefixed as “A”, “B”, and “C”.

Using each respondent's sentences as the unit of analysis, the coding team, made up of three coders (two males (OA, AG), one female (RK), all MDs), inductively and deductively identified codes and meaning units after an initial textual immersion [32]. A codebook was generated after analyzing the responses to the first 50 open-ended questions and the codebook was iteratively modified as the coding process continued (Table 2) [33]. Each coder coded the qualitative data pool independently and final codes were agreed upon through voice voting during coding and debriefing meetings. After an initial round of coding (open coding), the coding team performed focused coding, during which the initial codes were merged and re-categorized. Meaning units (exemplary sentence or phrasal codes) were generated from the sentences through the use of in-vivo, structural, and process coding techniques, and their counts were reported in tables [34]. Subthemes were identified by pooling codes with similar meaning units [32].

**Table 2:** Codebook

Theme	Description	Inclusion	Exclusion
Improved Knowledge	Improved or augmented comprehension, understanding, or command of serious illness conversations	Include any item that refers, explicitly or implicitly, to an individual’s improved knowledge in hospice and palliative care practice, with or without specific details	Exclude if the statement refers to the course and does not reflect on individual or group improved knowledge For implicit meaning: Exclude “close code but not exact” and “no, code is different” after applying the synonym rule
Improved Attitude	A positive feeling or disposition towards engaging in serious illness conversations	Include any item that refers, explicitly or implicitly, to an individual’s improved attitude in engaging in hospice and palliative care, with or without specifics	Exclude if the statement refers to the course and does not reflect on individual or group improved attitude. For implicit coding: Exclude “close code but not exact” and “no, code is different” after applying the synonym rule
Improved Practice	Improved day-to-day activities and expertise in engaging in serious illness conversations	Include any item that refers, explicitly or implicitly, to an individual’s improved practice or acquisition of skills in hospice and palliative care, with or without specific details	Exclude if the statement refers to the course and does not reflect on individual or group improved clinical practice or skill acquisition. For implicit coding: Exclude “close code but not exact” and “no, code is different” after applying the synonym rule

Synonym rule: For items that have implicit meanings, a synonym of the anchor word or phrase is applied and the sentence is re-assessed and categorized as either “yes, code is exact”, “no, code is different”, or “close code but not exact”

We employed several methods to ensure methodological and interpretive rigor. To ensure credibility, the coding team reported the final codebook created after a series of debriefing and coding meetings [35]. The open-ended questions that informed the responses provide information on the dependability of the study and the details of the study participants and the source of data provide information on the transferability of our findings [36]. By reporting the counts of the meaning units of each theme and using quotes from the participants to explain the thematic domain, we ensured the confirmability of the study [37].

### Human subject concern

Ethical approval was obtained from the New York University Grossman School of Medicine Institutional Review Board (ID: i18-00607) and the PRIM-ER study protocol is reported on *ClinicalTrials.gov* (NCT03424109) [38].

## Results

### Quantitative results: reach of intervention

There were a total of 1,029 EM providers eligible for the EM Talk training. These providers were predominantly aged 30—39 years (44%), male (51%), non-Hispanic White (77%), physicians (74%), with two to 10 years of practice (45%) (Table 3). A total of 879 out of 1,029 EM providers (85%) completed the EM Talk training (Table 4). The proportion of EM providers that had the training across the 33 EDs ranged from 63 to 100%. Between 2018 and 2020, a total of 2,698,198 unique patients, 66 years and older, visited the 33 EDs at least once. Of this population, the number (and proportion) of unique seriously ill patients (GAGNE score > 6) was 57,136 (2.1%). The yearly average of seriously ill patients across the 33 EDs was 19,045. We estimated that the trained EM providers would have encountered 16,389 seriously ill patients across all 33 EDs. Assuming a 100% practice rate among the trained EM providers, one trained EM provider will reach an average of 19 qualifying seriously ill patients and the number will vary from 4 to 115 across the 33 EDs.

**Table 3:** Demographic Characteristics of the Eligible EM Providers That Underwent the EM-Talk Training (*N* = 1,029)

Variables	Frequency (*N* = 1,029)
Age Categories	
Less than 30 years	77 (7.5)
30 – 39 years	455 (44.2)
40 – 49 years	294 (28.6)
50 – 59 years	144 (14.0)
60 years and older	59 (5.7)
Sex	
Male	528 (51.3)
Female	501 (48.7)
Race/Ethnicity	
Non-Hispanic White	792 (77.0)
Non-Hispanic Black	57 (5.5)
Hispanic	118 (11.5)
Other Races	62 (6.0)
Provider Type	
Physicians	762 (74.1)
Advanced Practice Provider	267 (25.9)
Years of Practice	
Less than 2 years	136 (13.2)
2 – 10 years	462 (44.9)
More than 10 years	431 (41.9)

**Table 4:** Reach of the *EM Talk* Training Across the Participating Emergency Departments

Hospital Name	Number of Full-Time EM Providers Trained	Total Number of Full-Time EM Providers	Percent Trained (%)	Average Annual Index Visits of Qualifying SI Patients	Number of SI Patients Encountered /Year	Yearly SI Patient: EM Provider ratio
Allegheny General Hospital	16	16	100.0	330	330	20.6
Baystate	33	35	94.3	915	863	26.2
Baystate Franklin	9	9	100.0	182	182	20.2
Beaumont Royal Oak	10	11	90.9	1265	1150	115.0
Beaumont Troy	15	18	83.3	1091	909	60.6
Bellevue Hospital Center	15	18	83.3	97	81	5.4
Brigham and Women's Hospital	19	21	90.5	1054	954	50.2
Brigham and Women's Faulkner	71	78	91.0	309	281	4.0
Christiana Hospital	33	44	75.0	892	669	20.3
Henry Ford Hospital	45	50	90.0	321	289	6.4
Henry Ford West Bloomfield	22	22	100.0	457	457	20.8
Henry Ford Fairlane	29	32	90.6	144	130	4.5
Hospital of the Univ of Penn	33	36	91.7	683	626	19.0
Mayo Austin Albert Lea	12	16	75.0	262	197	16.4
Mayo Mankato	17	22	77.3	367	284	16.7
Mayo St Mary	51	53	96.2	1162	1118	21.9
MD Anderson	21	26	80.8	1521	1229	58.5
Mount Sinai Beth Israel	16	19	84.2	281	237	14.8
Mount Sinai Hospital	47	48	97.9	722	707	15.0
Mount Sinai West	36	37	97.3	467	454	12.6
NYU Brooklyn	25	31	80.6	715	576	23.0
NYU Long Island	40	45	88.9	1100	978	24.5
Ochsner Medical Center	30	34	88.2	468	413	13.8
OSU Wexner Medical Center	49	78	62.8	800	502	10.2
Penn Presbyterian	15	20	75.0	305	229	15.3
Pennsylvania Hospital	10	13	76.9	280	215	21.5
UCSF Medical Center	15	18	83.3	623	519	34.6
UF Health Shands Hospital	23	31	74.2	215	160	7.0
UF Kanapaha	9	10	90.0	49	44	4.9
UF Springhill	11	13	84.6	141	119	10.8
University of Utah Hospital	35	39	89.7	490	440	12.6
Yale New Haven Hospital	33	42	78.6	1073	843	25.5
Zuckerberg SF General	34	44	77.3	264	204	6.0
**Total**	**879**	**1029**	**85.4**	**19,045**	**16,389**	**18.6**

Average SI Patients Qualifying Index Visits: Number of patients 66 years and older with an index ED visits who had a GAGNE index of six or higher. The average is calculated by dividing the 2018, 2019, and 2020 counts by 3. Estimated SI Patients Reached/Year = Percent Trained * SI Patients Qualifying Index Visits; Yearly SI Patient: *EM Provider ratio* Estimated SI Patients Reached/Number of EM Providers Trained, *OSU* Ohio State University, *UF* University of Florida, *UCSF* University of San Francisco, *NYU* New York University, *Univ of Penn* University of Pennsylvania

### Qualitative results: effectiveness of intervention

**Fig. 1:**
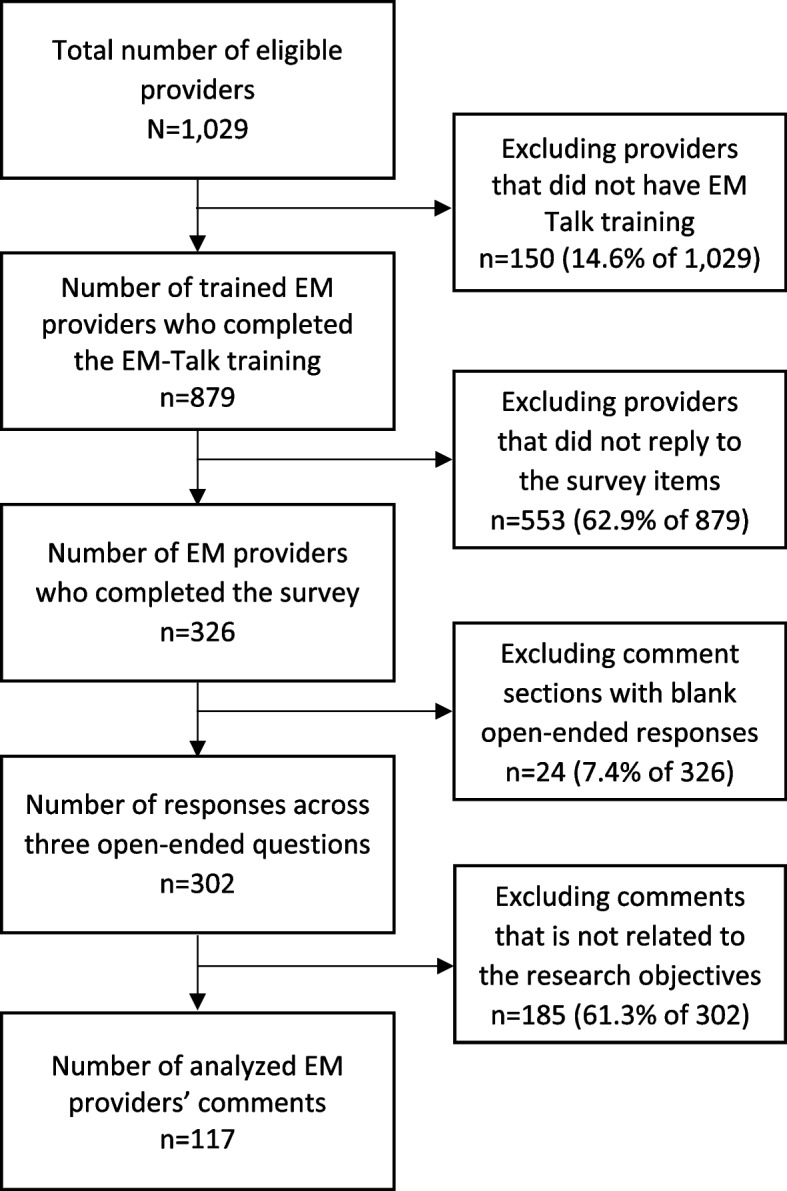
Data selection steps

Of the 879 EM providers who completed the EM Talk training, 326 completed the survey (37.1%) (Fig. 1). A total of 302 comments emerged from the open-ended question. After excluding 185 comments that were not related to either knowledge, attitude, or practice of SI conversations, we coded 117 open-ended responses (i.e. 38.7% of 302 comments). Sentences from 60 respondents were coded under the improved knowledge domain while sentences from 45 and 25 respondents were coded under the improved attitude and improved practice domains, respectively. With some sentences producing multiple codes across the thematic domains, the code counts exceeded 117 (Table 5).

**Table 5:** Content Coding

Theme and Subthemes^a^	Code Counts
**Improved Knowledge (*N* = 60)**^**b**^	
Acquired SI communication skills	47
Acquired general useful knowledge	14
**Improved Attitude (*N* = 45)**	
Attitudes toward engaging in SI conversations	30
Attitudes toward improving patient care	10
Attitudes toward receiving future training in SI conversations	5
**Improved Practice (*N* = 25)**	
Commitment to using acquired skills in clinical practice	20
Already utilizing taught skills in clinical practice	5

^a^Themes in bold; ^b^Multiple coding categories across subthemes account for the sum exceeding the total

### Improved knowledge

The theme of improved knowledge was referenced by 60 respondents. The most common subthemes that emerged from these responses were the acquisition of SI communication skills (*n* = 47) and acquired general useful knowledge (*n* = 14) (Table 6).

**Table 6:** Apriori themes, emerged subthemes, and the associated meaning units

Theme	Subtheme	Code label	Meaning Units
**Improved Knowledge**	Acquired SI communication skills	Acquired talking techniques in framing discussions	“Learned some techniques to talk to the family of palliative patients”
	Acquired useful general knowledge	Good learning experience	“This was a pretty good learning experience for me”
	Acquired empathy skills	Acquired empathetic skills	“…Learned a lot about empathetic skills that I can use in daily practice”
**Improved Attitude**	Attitude towards engaging in SI conversations	Comfortable and at ease	“…helped me become more comfortable and at ease with end-of-life conversations”
	Attitude towards improving patient care	I see the value	“I see the value it brings to patients and their families”
	Attitude towards receiving future training in SI conversations	Extremely applicable	“…it is extremely applicable to our practice. I would recommend all EM doctors undergo training such as this”
**Improved Practice**	Commitment to using acquired skills in clinical practice	I will incorporate skills into practice	“I look forward to incorporating this style of talking about goals of care with my patients and families”
	Already utilizing taught skills in clinical practice	I already used learned skill	“The very next day I had a patient/family interaction that I was able to identify and navigate because of the training…”

#### Acquired SI communication skills

A majority of respondents acknowledged that they *“learned some really valuable tools” (A254)* and that “*the tips and tricks provided were concise and therefore relatively easy to remember with regular practice/use” (A256).* One provider recounted:

“I did learn some helpful skills that I will try to bring into my practice.” (B64)

Some of the respondents were more specific on the tips and tricks they acquired which included using the “NURSE” statement (Naming, Understanding, Respecting, Supporting, and Exploring) for articulating empathy and the “REMAP” model (Reframe why the status quo is not working, Expect emotion and empathize, Map the future, Align with the patient’s values, and Plan medical treatment that match patient values) for addressing goals of care.

*“I will utilize NURSE and REMAP to help conversations.” (B47)*

*“I will practice more NURSE phrases and yet work to be much more direct.” (B48)*

*“I will utilize the REMAP structure.” (B75)*

To these trained EM providers, the SI communication skills taught in the course were viewed as “*techniques to talk to the family of palliative patients*” *(B35)*. One provider highlighted the importance of this skill based on the frequency of contact with SI patients and their caregivers in the EDs:

*“This was a useful educational intervention to ED providers who often have to have end-of-life discussions with patients and families in an emergent setting.” (B34)*

#### Acquired general useful knowledge

In contrast to EM providers that specified specific skills EM Talk provided, some providers reported a general improvement in their knowledge of palliative care. For some, the training was *“a pretty good learning experience for me” (C314)* while another provider felt the training *“really helped me grow as a provider” (A194)*. A provider shared:

*“I learned more than I thought I would, made me think about these issues more than I had before.” (B49)*

A few EM providers reported that the while the training *“did not introduce new concepts, it did help (me) put these concepts into an easier to deliver package”(B12).*

*“The experience was similar to what we did during residency but still allowed me to assess myself in a judgement free zone and identify areas where I still struggle. I came out with a couple of tips/tricks that I know I will incorporate into my practice moving forward.”(A179)*

### Improved attitude

The theme of improved attitude was present in 46 responses. The most common subtheme that was identified was improved attitudes toward engaging in hospice and palliative care discussions (*n* = 30). Less frequently identified subthemes included attitude towards improving patient care (*n* = 10) and attitude towards receiving future training on SI conversations (*n* = 5) (Table 6).

#### Improved attitude toward engaging in SI conversations

The improved attitude towards engaging in SI conversations referred to being “*more comfortable and at ease with end-of-life conversations” (A190).* For some EM providers, the training helped them “*realize the importance of having discussions with family early/often regarding goals of care for their loved ones*” (A188).

Some EM providers, however, discussed the deliberate attempt of the EM provider “*to slow down and listen to your patients and family members*” *(A166).* The importance of being intentional about listening was stated by one of the EM providers:

*“Patients end up being more satisfied when you listen and they feel as if their needs and concerns are being addressed” (C311)*

A few EM providers stated that the training helped increase the motivation to engage in SI conversations. For example, one provider wrote about negative past experiences and how the course made them feel more confident with such conversations:

*“…due to time constraints and some negative patient interactions regarding the goal of care discussions, I was initially resistant but now motivated and optimistic in my ability to navigate these talks” (B63).*

#### Improved attitude toward patient care

Improved attitude towards patient care referred to the EM providers *“see(ing) the value [the training] brings to patients and their families” (A208).* The training provided an opportunity for self-reflection and assessment with one provider stating that *“I identified various areas in which I can improve not only my communication in end-of-life discussions but also with all my patients*” (A258). The awareness of how the training may improve patient care served as a motivation for some EM providers to practice SI conversations.

*“[The course] pushed my comfort level with these discussions and has motivated me to practice and improve (B27).”*

#### Improved attitude towards future training on SI conversations

A few EM providers reflected on the EM Talk training and stated that *“[the training] is extremely applicable to our practice. I would recommend all EM doctors undergo training such as this” (A152).* Other EM providers referred to the effectiveness of the small group discussion format and the ability to download the VitalTalk app for future reference.

*“I had a great time in the small groups practicing difficult conversations. I also was happy to get the app downloaded to keep some very useful tools on hand” (B84).*

### Improved practice

The theme of improved practice was referenced by 25 respondents. The majority of these reflected the subtheme of commitment to using acquired skills in clinical practice (*n* = 20) while a minority of respondents (*n* = 5) stated that were already utilizing taught skills in clinical practice (Table 6).

#### Commitment to using acquired skills in clinical practice

The commitment to using acquired skills in clinical practice was indicated by providers who shared a plan to “*incorporating this style of talking about goals of care with my patients and families” (B63).* A provider acknowledged the ease of acquiring SI conversation skills and that it might take some time for the skill to become second nature.

*“It [the training] was interesting and the tool is easy to follow so it should be easy to incorporate into practice. I suspect it will be more comfortable with time and eventually become second nature” (A82).*

#### Already utilizing taught skills in clinical practice

A few EM providers expressed that, between the training completion and survey completion, they had been in clinical scenarios where they had to use some of the SI conversational skills taught. One provider stated that *“I feel better about approaching end-of-life discussions and have had some success in my recent practice” (B69).* Also, another provider attributed the success in navigating SI conversations he recently experienced to the training he received.

*“The very next day I had a patient/family interaction that I was able to identify and navigate because of the training” (C104)*

## Discussion

We report that across the 33 EDs enrolled in the PRIM-ER study, over 85 percent of the EM providers completed the EM Talk training and we estimate that these trained providers will reach approximately 16,389 seriously ill older adult patients that visit the ED. Also, across the thematic domains of improved knowledge, attitudes, and practice, the EM providers reported that the training improved their SI communication skills, improved their attitude towards engaging qualifying patients in SI conversations, and encouraged their commitment to using these learned skills in clinical practice.

The extensive reach of the EM Talk training is noteworthy. We trained a total of 879 EM providers, representing 85 percent of EM providers across the 33 EDs. The OncoTalk training by Back et al. [12], in comparison, reached 115 medical oncology fellows across 62 institutions, representing 42 percent of fellows across the selected institution. The GeriTalk intervention by Frydman et al. [18] reached 20 Geriatric and Palliative fellows across three institutions representing 100 percent of the fellows in the institutions. The extensive reach of the EM Talk training reflects the commitment of the departmental leadership of each site and their willingness to integrate the training into the educational curriculum in their departments. Also, compressing the training modules of the VitalTalk into a four-hour session made it logistically feasible to organize. In contrast, the OncoTalk [12] and GeriTalk training sessions [18] occurred over four days. In addition, our flexibility in converting the in-person training to a fully virtual training during the COVID-19 pandemic might have helped in logistically scheduling the sessions. Furthermore, we selected EM physician champions that were tasked with disseminating the information about the EM Talk training and facilitating attendance. The selection of appropriate and influential clinical champions is pivotal to the successful engagement and training of providers. Earlier studies have reported that clinical champions are instrumental in the quicker initiation of interventions, assist in overcoming institutional barriers, and can motivate and involve staff in clinical trials [39, 40].

We report that, assuming a 100 percent practice rate, a trained EM provider will reach between four and 115 seriously ill older adult patients every year depending on the ED volume, patient mix, geographic setting, and the type of acute and chronic diseases predominant among the population the ED serves, among other factors. This wide range of encounter highlights the diversity in the patient population that visit the ED, and the need for each ED to conduct a needs assessment, create ED-specific standard operating procedures in engaging qualifying patients in SI conversations, and provide a conducive environment for SI conversations in their respective EDs [41–44]. Engaging in SI conversations is never an easy task, and creating an enabling environment within the ED for EM providers to engage in such conversations may lighten the burden of delivering bad news and engaging patients in end-of-life goal discussions. Earlier studies have reported that some of the barriers EM providers and emergency nurses face in engaging qualifying patients in SI conversations include lack of privacy, limited patient engagement time, and the fast-paced ED work culture [9, 28, 45, 46].

EM Talk was designed to provide SI communication skills training to EM providers. Consistent with the goal of the intervention, the EM providers reported that they acquired SI communication skills, are willing to engage qualifying patients in SI conversations, and have the intent to incorporate these learned skills in clinical practice. The observed harmony between the expected goal and self-reported outcome may be explained by the evidenced-based pedagogical technique employed in delivering the EM Talk training. VitalTalk – the parent program from which EM Talk emerged has consistently prioritized role play and small group learning sessions as a bedrock of successful training sessions [10, 11]. Similarly, other authors that taught GeriTalk – another derivative of the VitalTalk, reported that Geriatric and Palliative Medicine fellows had high levels of satisfaction after they underwent the training [17, 18]. Similarly, Berg et al. [47] reported that Oncology fellows self-reported significant improvement in SI communication skills after undergoing OncoTalk training.

This study has its limitations. Although a large proportion of full-time EM providers completed the training, it is unlikely that all EM providers will embrace and utilize the SI communication skill in their practice. The estimated average of seriously ill patients that would be reached yearly, therefore, represents the best-case scenario. On the other hand, EM providers may learn from one another and the training and knowledge may even spread to those who are not formally trained– i.e., adoption of behavior due to peer influence. There is a possibility that attitude and practice towards engaging qualifying patients in SI conversations will differ by age, race/ethnicity, religious affiliation, and years of practice. Third, differences in the pedagogical styles of the different facilitators may positively or negatively influence the knowledge, attitude, and practice of EM providers toward engaging qualifying patients in SI conversations. Despite training 879 EM providers, about a third completed the open-ended question. The possibility exists that more meaning units might have emerged if everyone completed the survey. However, within the ambits of the responses obtained, the meaning units defined the bounded concepts of improved knowledge, attitude, and practice, and saturation was deemed achieved when no new information emerged from the codes. Also, the EM Talk course started as an in-person training program but was transitioned into an online training session due to the COVID-19 pandemic. We conducted a total of 106 EM Talk trainings. Before the start of the COVID-19 pandemic (May 2019-March 2020), we conducted 49 in-person trainings (46% of all trainings). At the start of the pandemic, we paused the intervention for six months (between March 2020 and September 2020). Following best-practice guidelines [48] and conversations with the leadership of each participating institution, we restarted the intervention in September 2020. Between September 2020 and December 2021, the remaining 57 sessions (54%) were virtual. It is unknown to what extent this change in pedagogy affects the reach and effectiveness of the intervention. Despite these limitations, this study is one of the few that assessed the effectiveness of EM Talk training across the domains of knowledge, attitude, and practice. Also, this is one of the few studies that used the RE-AIM framework to assess the reach and effectiveness of a provider-focused intervention. Furthermore, this study is strengthened by its spread across over 30 EDs and its large sample size.

## Conclusion

The EM Talk training reached a substantial proportion of EM providers working in the 33 EDs enrolled in the PRIM-ER study. The effectiveness of the EM Talk training was reflected across the thematic domains of improved knowledge, attitude, and practice evidenced by EM providers' self-reported acquisition of SI communication skills, willingness to engage qualifying patients in SI conversations, and intent to incorporate the learned skills into clinical practice. Future studies may assess to what extent learned communication skills translate into the proportion of qualifying seriously ill older adults with documented end-of-life goals and the proportion successfully transitioned to comfort care.

### Supplementary Information

**Additional file 1: Appendix 1.** Open-ended questions in the EM Talk post-training survey.

## Data Availability

The datasets used and/or analysed during the current study are available from the corresponding author on reasonable request.

## Abbreviations

RE-AIM: Reach, Effectiveness, Adoption, Implementation, and Maintenance
EM: Emergency Medicine
SI: Serious Illness
ED: Emergency Department
COREQ: Consolidated criteria for reporting qualitative research
PRIM-ER: Primary Palliative Care for Emergency Medicine
ELNEC: End-of-Life Nursing Education Consortium
CME: Continuing Medical Education
CMS: Centers for Medicare & Medicaid Services
KAP: Knowledge, Attitude, and Practice
MDs: Medical Doctor
