# Supplementary material for: Serious illness communication skills training for emergency physicians and advanced practice providers: a multi-method assessment of the reach and effectiveness of the intervention
Source: BMC Palliat Care. 2024 Feb 21;23:48. doi: 10.1186/s12904-024-01349-y (PMC10880358; doi:10.1186/s12904-024-01349-y)
Supplement: Supplementary file 1 — Additional file 1: Appendix 1. Open-ended questions in the EM Talk post-training survey. [file 12904_2024_1349_MOESM1_ESM.docx]

Appendix 1. Open-ended questions in the EM Talk post-training survey

| Question Number | Question | Response type |
| --- | --- | --- |
| 1. | What changes will you make to how you identify patients and/or family members who may be ready to discuss goals of care and/or palliative care options? | Open-ended |
| 2. | What changes will you make to how you counsel patients and/or family about end of life care? | Open-ended |
| 3. | What steps will you take to ensure that you involve all members of the ED care team to ensure a multi-disciplinary and team-based approach is used? | Open-ended |
| 4. | Did this activity meet the learning objectives as stated in the syllabus/handout? | Binary response (Yes/No) |
| 5. | Did this activity address factors beyond clinical care that affect the health of populations? | Binary response (Yes/No) |
| 6. | If yes, how? | Stemmed Question |
| 7. | In addition to the changes to practice described above, please list any other changes to practice you intend to make resulting from your participation in this activity. | Open-ended |
| 8. | Was this activity relevant to your practice? | Binary response (Yes/No) |
| 9. | Did this educational intervention fulfill your educational needs? | Binary response (Yes/No) |
| 10. | How could future activities address your needs better? | Open-ended |
| 11. | Are there any systemic barriers that would prevent you from making changes to practice? | Binary response (Yes/No) |
| 12. | Please select the option listed below that would provide the greatest systemic barrier to change:  * Formulary/coverage restrictions  * Time not allotted for implementation of new skills  lack of resources   - Insurance doesn't reimburse for treatment/etc. - Patient non-adherence - Organization does not support educational efforts - Policy issues within the organization - Other | Multiple choice: Each with a binary response (Yes/No) |
| 13. | Were the various learning formats (e.g., lecture, panel based discussion, Q&A) utilized in this activity appropriate for the achievement of the educational objectives listed? | Binary response (Yes/No) |
| 14. | Which of the options below would you have preferred as the main educational format for this educational intervention?   - Didactic Lecture - Case-based discussion with interactive ARS system - Q&A / Panel Discussion - Hands-On Training (simulation/cadaver) - Other | Multiple choice: Each with a binary response (Yes/No) |
| 15. | Disclosure of commercial support (if any) was clearly communicated. | Binary response (Yes/No) |
| 16. | Disclosure of relevant financial relationships of faculty were clearly communicated. | Binary response (Yes/No) |
| 17. | How can this information be clearly presented? | Open-ended |
| 18. | Faculty disclosed when they discussed unlabeled or unapproved uses of drugs or medical devices. | Binary response (Yes/No) |
| 19. | The activity was free of commercial bias. | Binary response (Yes/No) |
| 20. | Please give a detailed account, including the name of the presenter and nature of the perceived bias. | Stemmed Question |
| 21. | Please indicate what knowledge gaps, practice gaps, or patient health issues you have encountered in your own practice or in the profession that NYU SOM could address with continuing medical education initiatives. | Open-ended |
| 22. | Teaching methods/strategies/practices   - The instructors described the educational objective for his/her presentation clearly. - The instructors explained concepts clearly. - The instructors presented a balanced view of therapeutic options. - The instructors challenged my thinking on the subject matter. - The instructors encouraged questions and/or comments. - The instructors utilized presentation software effectively. - The instructors used other technology effectively. - The instructors provided useful educational materials | Each item with five-point Likert-based responses: strongly agree to strongly disagree |
| 23. | Which speaker(s) stood out amongst all of the speakers and why? | List-based response |
| 24. | In the space below, please reflect on your personal experience at this educational intervention. | Open-ended |
| 25. | Did you utilize our course App and the resources provided to you within the App? | Binary response (Yes/No) |
| 26. | Which resources did you utilize? | List-based response |
| 27. | Did this deepen and strengthen your learning? | Binary response (Yes/No) |
| Data for the qualitative analysis was from one of the open-ended questions in the EM Talk post-training survey – designed consistent with the requirement of continuing medical education assessment.  ED: Emergency Department; Q&A: Question and Answer; ARS: Audience Response System; NYU SOM: New York University School of Medicine; App: Application | | |
